# Elimination of *Candida* Sepsis and Reducing Several Morbidities in a Tertiary NICU in Greece After Changing Antibiotic, Ventilation, and Nutrition Protocols

**DOI:** 10.3390/antibiotics14020159

**Published:** 2025-02-05

**Authors:** Niki Dermitzaki, Natalia Atzemoglou, Vasileios Giapros, Maria Baltogianni, Dimitrios Rallis, Theodoros Gouvias, Anastasios Serbis, Aikaterini Drougia

**Affiliations:** 1 Neonatal Intensive Care Unit, School of Medicine, University of Ioannina, 45500 Ioannina, Greece; n.dermitzaki@uoi.gr (N.D.); md06684@uoi.gr (N.A.); mbalt@doctors.org.uk (M.B.); drallis@uoi.gr (D.R.); gouviasteo@yahoo.gr (T.G.); katdrougia@gmail.com (A.D.); 2 Pediatric Department, School of Medicine, University of Ioannina, 45500 Ioannina, Greece; aserbis@uoi.gr

**Keywords:** late-onset sepsis, preterm neonate, neonatal intensive care, antibiotic stewardship, invasive candidiasis

## Abstract

Background/Objectives: In recent years, strategies for improving outcomes in preterm neonates have been implemented in various aspects of neonatal care. This study aims to determine the prevalence, microbiology, and outcomes of late-onset sepsis (LOS) and the incidence of other morbidities in very preterm neonates following the implementation of specific infection control, enteral feeding, and ventilation strategies. Methods: This study retrospectively compared the morbidity and mortality of preterm neonates with a 23–32 weeks gestational age over two periods, period A (2010–2014),and period B (2018–2022). A series of changes were introduced between these periods, including restrictive use of antibiotics, aggressive enteral feeding, and wider use of non-invasive ventilation modalities. Results: A total of 310 neonates were included: 163 in period A and 147 in period B. The mean duration of antibiotic treatment was reduced from 4 ± 2 to 2 ± 1 days and from 5 ± 2 to 3 ± 1 days for suspected early-onset sepsis and LOS, respectively, and from 11.2 ± 4 to 16 ± 4 days for confirmed LOS between the two study periods. The incidence of LOS was 24% and 18%, while, for multiple LOS episodes, it was 26% and 11% in periods A and B, respectively. Total parenteral nutrition (TPN) duration and gestational age were independent predictors of LOS in both periods. The rate of *Candida* infections declined from 9.2% to 0.7%. The full enteral nutrition in period B was achieved after a median of 7.5 days compared with 10 days (*p* = 0.001), resulting in fewer days of TPN (*p* = 0.008). Episodes of feeding intolerance and necrotizing enterocolitis I (NEC I) were significantly reduced (*p* < 0.001). Incidence of intraventricular hemorrhage were significantly decreased. Conclusions: After changing antibiotic, ventilation, and nutrition protocols, *Candida* infections were almost completely eliminated. The incidence of LOS and multiple LOS episodes decreased. Early full enteral nutrition was achieved without adverse effects, and fewer episodes of food intolerance were observed. *Candida* elimination appears feasible when antibiotic stewardship is implemented in conjunction with other interventions in an NICU.

## 1. Introduction

Preterm birth represents a significant global health concern, affecting approximately 10% of all pregnancies [1]. Prematurity is a major cause of morbidity and mortality in the neonatal population, with an increased risk of short- and long-term complications [2]. Among preterm neonates, 15% are born at a gestational age of less than 32 weeks and constitute the most vulnerable population [1].

Improving outcomes and reducing morbidity in preterm neonates represent a considerable challenge, particularly in recent years, given the increase in the survival of preterm neonates at younger gestational ages. It is acknowledged that a holistic approach is necessary while caring for preterm neonates in the neonatal intensive care unit (NICU). In recent decades, neonatologists have focused on implementing new strategies regarding respiratory support, enteral feeding promotion, infection control, and antibiotic reduction in NICUs to optimize neonatal care and outcomes. Implementing these practices has improved morbidity and mortality outcomes, but more studies are needed to provide more robust evidence [3,4,5,6].

Despite improvements in NICU care, late-onset sepsis (LOS) remains a significant threat to preterm neonates, with prevalence increasing with decreasing gestational ages [7,8]. The potentially detrimental consequences in terms of morbidity and mortality, in conjunction with the non-specific presentation and the low sensitivity of diagnostic biomarkers, often lead to the overprescription and administration of unnecessarily prolonged courses of broad-spectrum antibiotics [9,10,11]. The emergence of multidrug-resistant strains is an inevitable consequence of the excessive use of broad-spectrum antibiotics [9]. Furthermore, an increasing number of studies have demonstrated an association between prolonged antibiotic use in preterm neonates and adverse outcomes, including late-onset sepsis (LOS), intraventricular hemorrhage (IVH), necrotizing enterocolitis (NEC), bronchopulmonary dysplasia (BPD), retinopathy of prematurity (ROP), and mortality [12,13,14,15]. Therefore, rational and appropriate use of antibiotics in terms of administration criteria, antimicrobial agent choice, and therapy duration is necessary. The implementation of antimicrobial stewardship programs in NICUs aims to promote optimal antibiotic use and minimize short and long-term adverse effects in the neonatal population [16,17].

A significant concern associated with prolonged antibiotic exposure in very-low-birth-weight neonates is the increased risk of invasive candidiasis, particularly in cases when broad-spectrum antimicrobial agents, such as third-generation cephalosporins and carbapenems, are administered [18,19]. The reduction in broad-spectrum antibiotic use in NICUs has been associated with a decreased incidence of invasive candidiasis, particularly in extremely low-birth-weight neonates [4,18].

In recent years, following an administrative change, a battery of interventions have been made in this NICU to optimize the outcomes for very preterm neonates by implementing new strategies regarding nutrition, ventilation, and antibiotic administration. We hypothesize that these changes and interventions have led to improved outcomes compared to an earlier period. This study aims to determine the prevalence, microbiology, and outcomes of LOS and the incidence of other morbidities in very preterm neonates following the implementation of certain infection control practices, aggressive enteral feeding, and the wider use of newer ventilation modalities.

## 2. Results

A total of 342 preterm neonates with a gestational age of 23 to 32 weeks were admitted to the NICU during the two study periods. Of these, 28 were excluded due to early postnatal death (16 in period A, 12 in period B) and 4 were excluded due to major congenital anomalies (one in period A, three in period B). The study included 310 neonates, with 163 being admitted to the NICU in period A and 147 in period B.

### 2.1. Neonatal and Obstetrics Characteristics

The gestational age and the birth weight of neonates in period A and period B were not significantly different (29.0 ± 2.2 vs. 29.3 ± 2.3 weeks and 1198 ± 325 vs. 1220 ± 367 g, respectively) (Table 1). There was no difference between the two periods in the proportion of neonates born to multiple pregnancies (44% vs. 42%, *p* = 0.73) or through assisted reproductive technologies (32% vs. 35%, *p* = 0.51). Antenatal steroid use increased significantly between the two periods, with administration in 70% of pregnancies in period A and 84% in B (*p* = 0.03). Vaginal births were less common in period B (14% compared to 25% in period A, *p* = 0.02). Maternal chorioamnionitis was diagnosed in significantly more cases in period B (*p* < 0.01).

### 2.2. Antibiotics

The main changes in antibiotic type and duration between the two periods were the following: the duration of antibiotic treatment in suspected EOS was reduced from a mean of 4 ± 2 to 2 ± 1 days, while, in suspected LOS, it was reduced from 5 ± 2 to 3 ± 1 days, respectively. In cases of possible sepsis, the mean duration of antibiotics decreased from 6 ± 2 days in period A to 3.5 ± 1.5 days in period B. The mean duration of treatment for LOS was 11.2 ± 4 days compared with 16 ± 4 days in the first period (Table 2). The antibiotics used in the second period were gentamicin and ampicillin as the first line and teicoplanin with piperacillin/tazobactam or ciprofloxacin or amikacin as the second or even third line (on rare occasions). The use of third or higher generations of cephalosporins, linezolid, or carbapenems that were used during the first study period was gradually totally eliminated.

### 2.3. Late-Onset Infections

In period A, the incidence of LOS was 24% (39 out of 163), while, in period B, 18% (27 out of 147). Multiple LOS episodes were experienced by 26% of neonates (10 of 39) in period A and 11% (3 of 27) in period B (Table 3). The gestational age of neonates with LOS was not significantly different in the two periods (*p* = 0.52). The highest incidence of LOS was observed in neonates with a gestational age of less than 28 weeks: 42.3% (22/52) in group A and 33% (14/43) in group B. The rate of CLABSIs was not significantly different between the two periods, with 30% of LOS episodes in period A and 22% in period B (*p* = 0.39). The causative pathogen distributions in period A vs. period B were as follows: CoNS 46% vs. 52% (*p* = 0.65), other Gram-positive bacteria 13% vs. 12% (*p* = 0.92), Gram-negative bacteria 33% vs. 42% (*p* = 0.42), and fungi 39% vs. 4% (*p* < 0.001) (Table 3). The overall rate of fungal infections in the very preterm population was 9.2% in period A and 0.7% in period B. All fungal infections in both study periods were caused by *Candida* spp. In period A, the most prevalent *Candida* strain isolated was *Candida albicans* (7/15), followed by *Candida lusitaniae* (4/15), *Candida parasilosis* (4/15), and *Candida glabrata* (1/15). In period B, the sole case of *Candida* sepsis was attributed to *Candida albicans*.

Mortality attributed to LOS was 23% in period A (5 g-negative, 4 fungal) and 22% in B (6 g-negative sepsis). In period B, the sepsis-related mortality rate in neonates above 28 weeks was found to be 0% (0/13), in contrast to the 29.4% (5/17) observed in the same gestational age group in period A.

### 2.4. Enteral Feeding

A significant discrepancy was observed between the two time periods with regard to the time of attainment of full enteral feeding (Table 2). In period A, full enteral nutrition was achieved after a median of 10 days. In contrast, in period B, it was achieved after a median of 7.5 days (*p* = 0.001), resulting in fewer days of parenteral nutrition, reaching a median of 10 days in period A and 7 days in period B (*p* = 0.008). This is due to the limited duration of trophic feeding in period B and the more rapid increase in milk volume (30–40 mL/kg/day). Parenteral nutrition was discontinued when the milk volume reached 100–120 mL/kg, and the increase was continued to a final volume of 200–240 mL/kg. Fortified breastmilk or preterm formula, if breastmilk was not available, was used in both study periods. There was no significant difference in the type of milk given between the two study periods. Despite the more aggressive enteral feeding, episodes of feeding intolerance were significantly lower in period B (*p* < 0.001). Episodes of NEC I significantly decreased in period B (*p* < 0.001); however, in regard to NEC grade II and III, no significant difference was observed.

### 2.5. Respiratory Support

In period A, significantly more neonates received invasive mechanical ventilation (79% vs. 66%, *p* = 0.009), and the mean duration of mechanical ventilation was longer (3 vs. 1 day, *p* = 0.018). No significant difference was observed in the proportion of neonates that subsequently developed BPD (Table 2).

### 2.6. Neonatal Morbidities and Mortality

A significantly lower proportion of neonates developed IVH of any grade in period B, 27%, compared to 46% in period A (*p* < 0.001) (Table 2). This difference remained significant, although less pronounced, for neonates who developed severe IVH (grade III-IV) (6% in period B vs. 13% in period A, *p* = 0.49). The proportion of neonates diagnosed with cystic periventricular leukomalacia (cPVL) remained unchanged (*p* = 0.19). Retinopathy of prematurity of any grade and ROP 3 were diagnosed in 15% and 3% of neonates in period A and 18% and 6% in period B, respectively. The overall mortality rate of very preterm neonates, although lower in the second period, did not reach statistical significance, with rates of 11% (18/163) and 8% (12/147) reported in periods A and B, respectively (*p* = 0.39) (Table 3).

In a multiple regression analysis od each study period, the LOS status (yes or no LOS) was the dependent variable, and the independent variables were the gestational age, birth weight, duration of mechanical ventilation, duration of antibiotics, number of red blood cell transfusions, age of attainment for full enteral feeding, duration of TPN, length of stay in the NICU, presence of BPD, and type of feeding. The analysis showed that the only predictors of LOS in both study periods were the duration of TPN (OR: 1.12, CI: 1.06, 1.19) and gestational age (OR: 0.84, CI: 0.8, 0.9), while feeding with human milk was also an independent predictor in the second study period (OR: 0.25 CI: 0.1, 0.6).

## 3. Discussion

The present retrospective comparative study examines the prevalence, microbiology, and outcomes of LOS and the incidence of other neonatal morbidities in very preterm neonates over two periods following changes in preterm care in the NICU regarding antibiotic use, enteral feeding, and ventilation techniques. Regarding LOS, the duration of TPN and gestational age were the only independent predictors.

The prevalence of LOS among very-low-birth-weight neonates was observed to be 24% and 18% in periods A and B, respectively. This rate is comparable to that previously reported by other centers in preterm populations [20,21,22,23]. There was a notable reduction, although not one that was statistically significant, in the proportion of newborns with more than one episode of LOS, decreasing from 26% to 11%. The vicious circle of antibiotic use in preterm neonates, whereby the administration of antibiotics in the early stages is linked to subsequent infections, has been described previously [13,24]. In the second period of this study, the use of empirical antibiotics was significantly restricted. The decision to administer antibiotics for EOS was based on risk categorization in accordance with the guidelines published by the American Academy of Pediatrics in 2018 [25]. Furthermore, when antibiotic treatment was initiated based on risk factors or clinical presentation, it was discontinued after 36–48 h if cultures were negative and the clinical condition was reassuring. The implementation of these strategies resulted in a significant reduction in the duration of empirical antibiotics, from a mean of 4 ± 2 to 2 ± 1 days for suspected EOS and from a mean of 5 ± 2 to 3 ± 1 days for suspected LOS in periods A and B, respectively. In a multicenter study involving 25 NICUs in China, Yu et al. demonstrated that the administration of antibiotics during the first week of life in preterm neonates without culture-proven sepsis resulted in approximately fivefold increased odds of antibiotic use later during the NICU stay. In prolonged or wide-spectrum antibiotic administration, the risk increased by 6–8.5 times compared to neonates that did not receive antibiotics [13].

The use of broad-spectrum antibiotics is associated with alterations in the intestinal microbiota in terms of species diversity, metabolic functions, and growth, and it negatively impacts the maturation process of the gastrointestinal tract, particularly in preterm neonates. It has been demonstrated that the adverse impact of antibiotics on gut microbiota during the first postnatal weeks is more pronounced and persistent and that the dysbiosis persists after antibiotic discontinuation. This is further compounded by prolonged use and the administration of broad-spectrum antibiotics. Intestinal microbiota exerts a multidimensional effect, and imbalances significantly impact general health. In neonates, dysbiosis has been associated with an increased incidence of short-term morbidities such as LOS, NEC, BPD, and ROP and long-term morbidities such as neurodevelopmental impairment, obesity, and allergic diseases [17,26,27].

An important finding of this study was the almost complete elimination of *Candida* sepsis in the NICU after implementing the strategies described above. Invasive candidiasis is a major cause of concern for the preterm population, with a high rate of morbidity and mortality that is inversely correlated with gestational age and birth weight [28,29]. In a cohort of extremely preterm neonates, the mortality rate was as high as 50%, with almost half of survivors experiencing severe neurodevelopmental impairment [23]. In the first period of the present study, the mortality rate for *Candida* sepsis was 27%. Between the two periods of the study, the rate of invasive *Candida* infections in the very preterm population declined from 9.2% to 0.7%. Several factors are likely to have contributed to this significant decline, including the restrictive use of antibiotics, the limited duration of parenteral nutrition, the reduction in the use of invasive ventilation, and the enhancement of sterile precautions during central line management. Notably, the practices regarding antifungal prophylaxis in high-risk neonates remained unchanged throughout the two periods of the study.

In the second period of this study, the strategies implemented regarding the use of antibiotics, with shorter duration and restrictive use of broad-spectrum antibiotics, were expected to reduce the incidence of *Candida* infections. Antibiotics suppress the normal bacterial flora of the gastrointestinal tract, thus diminishing the competitive effect that would naturally inhibit *Candida* proliferation [15]. Aliaga et al., in a large multicenter study, observed a 2.9–7.3% reduction in invasive candidiasis in very-low-birth-weight neonates for every 10% reduction in broad-spectrum antibiotics [4]. The average use of antibiotics per neonate in a NICU, particularly third-generation cephalosporins and carbapenems, has been shown to correlate with the prevalence of systemic *Candida* infections [18,30]. The use of broad-spectrum antibiotics, including third-generation cephalosporins and carbapenems, was largely reduced during the second study period.

In addition to the restrictive use of antibiotics, the reduced duration of parenteral nutrition may have contributed to the elimination of *Candida* infections in the second period of this study. Parenteral nutrition is a recognized risk factor for systemic candidiasis in neonatal populations [31,32,33,34]. The prolonged need for vascular access, either by CVC or peripheral venous cannula, results in penetration of epithelial barriers and facilitates *Candida* invasion [29,35]. In addition, the formation of biofilms in implanted medical devices is an important aspect of the pathogenesis of systemic candidiasis. Fungi in these structures are protected from antifungal drugs and the host immune response, and biofilms act as reservoirs for fungal dissemination [36,37]. However, in a previous prospective cohort study, the association between parenteral nutrition and candidiasis was independent of CVC use [33]. Contamination during the preparation of parenteral nutrition and the known effect of lipid emulsions in promoting *Candida* proliferation, as well as the ability to form biofilms, are potential contributors to the association between parenteral nutrition and candidiasis [38,39].

Another factor that could potentially contribute to the elimination of *Candida* infections is the reduced use of mechanical ventilation in the second period of the study. In addition to the recognized risk of lung injury and the increased risk of chronic lung disease, invasive ventilation disrupts epithelial barriers and predisposes to bacterial and fungal infections [35]. A significant proportion of preterm neonates require respiratory support during the initial postnatal period, which can extend over a range of durations. In recent decades, significant progress has been made in the field of respiratory support through the widespread use of non-invasive ventilation modalities [40,41]. In the second period of the study, the employment of non-invasive ventilation techniques was broadened. This facilitated either the avoidance of mechanical ventilation or earlier extubation.

In recent years, a growing body of evidence has indicated that the early initiation and more rapid advancement of enteral feeding, even in extremely low-birth-weight neonates, are beneficial and not associated with increased risk of NEC or other adverse outcomes [42,43,44,45,46,47]. In the second period of the present study, more aggressive feeding protocols were implemented. These feeding protocols consisted of early initiation and rapid volume advancement, resulting in full enteral feeding achievement at a median of 7.5 days, in contrast to the 10 days in the first period. As anticipated, the attainment of full enteral feeding at an earlier postnatal age resulted in enhanced growth, as evidenced by the significant reduction in the incidence of extrauterine growth restriction. A number of studies have demonstrated a correlation between the administration of parenteral nutrition and its duration with LOS in preterm neonates [48,49,50,51]. In the present study, the duration of TPN was identified as the sole independent predictor of LOS in both periods, in addition to gestational age.

In period B, the incidence of suspected NEC (NEC I) decreased significantly compared to period A. This can be at least partly attributed to the restriction of early antibiotic use. The intestinal microbiota of preterm infants has been demonstrated to be more susceptible to disruption by exogenous factors such as early antibiotic exposure [52,53,54]. A number of studies have evidenced an association between the duration of early antibiotics and NEC [24,55,56,57,58]. A recent population-based study of very preterm neonates with no culture-proven sepsis reported that early prolonged empirical antibiotic administration (>5 days) was associated with a more than twofold increase in the odds of developing severe NEC [59]. In the current study, the duration of empirical antibiotics in neonates with suspected EOS and LOS was reduced significantly. However, no decrease in the incidence of surgical NEC was noted.

A significant reduction in IVH of any grade was observed in the latter period of the study. The difference in the incidence of IVH between the two periods remained significant, although to a lesser extent when comparing severe IVH. The reduced prevalence of IVH is likely attributable to a combination of factors. In a recent study, Korcek et al., in a large cohort of very preterm neonates, identified the administration of antenatal steroids and the use of cesarean sections as critical factors in the prevention of IVH [60]. In period B of this study, a higher proportion of neonates fulfilled the abovementioned factors compared to period A. In addition, intubation procedures and mechanical ventilation during the first postnatal days have been suggested as potential risk factors for IVH, as they may be associated with fluctuations in cerebral blood flow [61]. However, the observed discrepancy between the two study periods cannot be attributed to a single factor. Instead, it may be attributed to an overall improvement in the perinatal care of preterm neonates, with a number of measures being introduced into everyday clinical practice.

An increase in the incidence of ROP of any grade and ROP 3 was observed between period A and period B. This is hypothesized to be due to an increased proportion of neonates with a gestational age of <26 weeks, which represent the higher risk population, in period B compared to period A (11.5% and 7.3%, respectively).

This study has some limitations. The main limitations are its retrospective nature and that it is a single-center study with a relatively modest sample size. Consequently, it is challenging to generalize the results. Furthermore, the impact of other changes in daily practice regarding the care of preterm neonates throughout the study period cannot be excluded. Such changes include intensifying infection precautions, the availability of more technologically advanced equipment, and a marginally higher nurse-to-neonate ratio.

## 4. Materials and Methods

### 4.1. Study Population

The medical records of all preterm neonates with a gestational age of 23–32 weeks admitted to the NICU of the University Hospital of Ioannina between 2010 and 2014 (period A) and 2018–2022 (period B) were retrospectively reviewed for eligibility for inclusion in the study. This is a regional level III NICU covering the entire neonatal population of Northwestern Greece, with 400–450 admissions per year. All very preterm neonates who survived beyond the third postnatal day and had no major congenital anomaly were included in the study. Pregnancy details and neonatal and clinical characteristics from admission to the NICU to hospital discharge were collected for all participants in the study. The study was conducted in accordance with the Declaration of Helsinki and was reviewed and approved by the Institutional Scientific Review Board at Ioannina University Hospital. Informed consent was obtained from the parents of all neonates involved in the study.

The primary outcome of this study was to determine the prevalence, microbiology, and outcomes of LOS in very preterm neonates following the implementation of certain infection control practices, aggressive enteral feeding, and the wider use of newer ventilation modalities. Secondary outcomes included the incidence of other morbidities such as intraventricular hemorrhage, bronchopulmonary dysplasia, and retinopathy of prematurity.

### 4.2. Definitions

LOS was defined as a positive blood, urine, and/or cerebrospinal fluid culture beyond 72 h of age. For *coagulase*-negative *Staphylococcus* (CoNS) infections to be defined as LOS, two positive cultures and clinical signs of sepsis were required. Pathogens isolated from cultures were categorized into Gram-negative bacteria, Gram-positive bacteria, CoNS, and fungi. If the same pathogen was isolated in repeated cultures within 7 days, a single episode of sepsis was counted. Suspected sepsis was defined as the presence of clinical signs (clinical sepsis) in the absence of a positive sterile site culture and elevated inflammatory markers. Possible sepsis was considered in cases with elevated inflammatory markers but negative cultures and a reassuring clinical condition. A central-line-associated bloodstream infection (CLABSI) was defined as the isolation of a pathogen from the blood culture of neonates with a central line at the time of infection or within the previous 48 h [62].

The following classification systems were used for the different neonatal morbidities. The severity of IVH was graded according to the Volpe classification [63]. The diagnosis of BPD was based on the need for supplemental oxygen or respiratory support at 36 weeks postmenstrual age for at least three consecutive days to maintain an arterial oxygen saturation of 90–95%, according to the revised National Institute of Child Health and Human Development (NICHD) definition [64]. NEC was classified based on the modified Bell criteria [65]. ROP was classified according to the International Classification of Retinopathy of Prematurity [66]. A neurological examination was conducted on discharge using the Hammersmith Infant Neurological Examination (HINE) [67].

### 4.3. Differences in Practice Between the Two Time Periods

In the current study, the morbidity and mortality of preterm neonates < 32 weeks of gestational age were retrospectively compared between two time periods. The two periods were 2010–2014 (period A) and 2018–2022 (period B). The decision to compare these two periods was made because, following period A, after an administration change in the NICU, a series of changes in the care of very preterm neonates were introduced concomitantly in terms of antibiotics administration, nutrition, and respiratory support.

In period B, antibiotics with a narrow spectrum and reduced duration were administered. Specifically, in the presence of risk factors for early-onset sepsis (EOS) or suspected LOS, antibiotic treatment was discontinued after 36–48 h following sterile blood cultures. In neonates with possible sepsis where, despite the sterility of the blood cultures and the reassuring clinical condition, the inflammatory markers were elevated, the duration of antibiotic administration was individualized, taking into account the level of inflammatory markers and the presence of a risk factor for sepsis. In period B, the duration of treatment for bloodstream infections was 10 to 14 days. For uncomplicated meningitis, antibiotics were administered for 14 days if Gram-positive bacteria were isolated and for 21 days if Gram-negative bacteria were isolated. The first-line antibiotics in period B were ampicillin and gentamicin. Ampicillin was administered at a dose of 100–300 mg/kg/day, divided into 2–3 slow push doses depending on postmenstrual age and indication. Gentamicin was infused over 30 min, and the dose was selected according to postmenstrual age, ranging from 4 mg/kg/day to 5 mg/kg/48 h in the youngest neonates. Teicoplanin, piperacillin/tazobactam, amikacin, or ciprofloxacin were used as second/third line. Third or higher-generation cephalosporins, linezolid, or carbapenems, which were used during period A, were either extremely reduced or eliminated.

From 2016 to 2019, this NICU participated in a national collaboration called Preventing Hospital Infections in Greece (PHIG), which aimed to reduce the rate of CLASBIs [62]. In the context of this collaboration, in addition to antibiotic stewardship, a CLASBI prevention bundle was implemented into the daily routine to enhance hand hygiene, sterile precautions during central line insertion and care, and early removal. These strategies were incorporated into daily neonatal care in the NICU and continue to be applied.

With regard to enteral feeding of very preterm neonates in period A, trophic feeding was maintained for five days, followed by a slow advance of 20 mL/kg/day. In period B, more aggressive feeding protocols were followed. Trophic feeds (20 mL/kg) were maintained for three days in neonates with a birth weight (BW) below 1250 g or for one day in neonates with BW > 1250 g. Following trophic feeding, there was a more rapid increase in milk volume, by 30–40 mL/kg/day, depending on birthweight, to a volume of 200–240 mL/kg [68]. Fortified breastmilk was the preferred choice for feeding, but, if insufficient or unavailable, preterm infant formula was given.

During period A, mechanical ventilation was more often used for respiratory support for preterm neonates. Conversely, during period B, the systemic utilization of non-invasive ventilation techniques, including nasal intermittent positive pressure ventilation (NIPPV), nasal continuous positive airway pressure (nCPAP), and heated humidified high-flow nasal cannula (HHHFNC), enabled either the avoidance of mechanical ventilation or facilitated early extubation.

### 4.4. Statistical Analysis

The comparisons between the two study groups of neonates with or without LOS were made, respectively, either by the *t*-test or with a Mann–Whitney U–test after evaluation of parameters for normal or not normal distribution. The Benjamini–Hochberg procedure to adjust for the multiple comparisons was also used. A *p* < 0.05 was considered significant, while values are expressed as mean value ± standard deviation (SD) or as median and interquartile range (IR). A multiple regression analysis and logistic regression were undertaken to find possible independent parameters related to the presence of LOS. All the parameters that entered the model were tested for collinearity. The Stat View software 5.0 application of SAS Institute Incorporation (Cary, NC, USA) was used [69].

## 5. Conclusions

The almost complete elimination of *Candida* infections, a significant threat to preterm neonates in the NICU, has been the most significant impact of a series of changes in this NICU. Moreover, a decline in the incidence of neonates with multiple episodes of LOS was observed. The early attainment of full enteral nutrition was achieved without adverse effects, and fewer episodes of feeding intolerance were observed. The results of the current study support the idea of a holistic approach to the care of preterm neonates and further support the application of such changes in an NICU environment.

This study also supports the notion that the promotion of restrictive use and the administration of narrow-spectrum antibiotics is facilitated by targeted antibiotic therapy and early cessation of therapy in cases of unproven sepsis. This can potentially minimize the risks of irrational antibiotic use in this vulnerable population. Moreover, it is apparent that the early initiation of enteral feeding in very preterm neonates and its subsequent rapid advancement, among other benefits, minimizes the risks associated with prolonged parenteral nutrition. All the above elements combined makes the reducing of invasive *Candida* infections in a current NICU environment feasible.

## Figures and Tables

**Table 1:** Comparison of obstetric and neonatal characteristics of preterm neonates in the two study periods.

Parameters	Period A (n = 163)	Period B (n = 147)	*p*
Sex, male	75 (46%)	83 (57%)	0.06
Gestational age, weeks	29.0 ± 2.2	29.3 ± 2.3	0.35
Birthweight, grams	1198 ± 325	1220 ± 367	0.56
SGA neonates	19 (12%)	25 (17%)	0.17
Multiparity	72 (44%)	62 (42%)	0.73
Maternal age	32.2 ± 5.7	33.7 ± 5.8	0.02
IVF	52 (32%)	52 (35%)	0.51
Antenatal steroids	114 (70%)	124 (84%)	0.003
Maternal chorioamnionitis	2 (2%)	21 (15%)	<0.001
PROM	43 (26%)	43 (29%)	0.57
Mode of delivery, vaginal	40 (25%)	20 (14%)	0.02
Inborn	148 (91%)	135 (92%)	0.74

SGA: small for gestational age; IVF: in vitro fertilization; PROM: premature rupture of membranes.

**Table 2:** Neonatal characteristics of preterm neonates and outcomes in the two study periods.

Parameters	Period A (n = 163)	Period B (n = 147)	*p*
Full enteral feeding	10 (6, 14)	7.5 (5, 10)	0.001
Breast milk	29 (28%)	43 (31%)	0.57
Feeding intolerance	61 (37%)	20 (14%)	<0.001
TPN duration, days	10 (5, 22)	7 (5, 14)	0.008
Extrauterine growth restriction	88 (61%)	61 (45%)	0.01
Duration of antibiotics in suspected EOS	4 ± 2	2 ± 1	0.001
Duration of antibiotics in suspected LOS	5 ± 2	3 ± 1	0.001
Duration of antibiotics in LOS	16 ± 4	11.2 ± 4	0.001
Duration of antibiotics in possible sepsis	6 ± 2	3.5 ± 1.5	0.0001
Central venous catheter	50 (31%)	57 (39%)	0.13
NEC	68 (42%)	29 (20%)	<0.001
NEC III	3 (2%)	2 (1%)	0.72
Ventilation duration, days	3 (0.1, 14)	1 (0, 7.5)	0.01
Invasive ventilation	129 (79%)	97 (66%)	0.009
BPD	37 (26%)	26 (19%)	0.18
RBC transfusions at 28 days	1 (0, 2)	1 (0, 2.5)	0.70
ROP	22 (15%)	24 (18%)	0.59
ROP 3	4 (3%)	8 (6%)	0.20
IVH	74 (46%)	38 (27%)	<0.001
IVH III-IV	21 (13%)	9 (6%)	0.04
cPVL	6 (4%)	2 (2%)	0.19
Neurological discharge, normal	133 (92%)	120 (90%)	0.53
Mortality	18 (11%)	12 (8%)	0.39

TPN: total parenteral nutrition; EOS: early-onset sepsis; LOS: late-onset sepsis; NEC: necrotizing enterocolitis; BPD: bronchopulmonary dysplasia; RBC: red blood cell; ROP: retinopathy of prematurity; IVH: intraventricular hemorrhage; cPVL: cystic periventricular leukomalacia.

**Table 3:** Comparison of sepsis prevalence and microbiology in late-onset sepsis in preterm neonates between the two study periods.

Parameters	Period A (n = 163)	Period B (n = 147)	*p*
EOS	4 (2.5%)	1 (0.7%)	0.22
LOS	39 (24%)	27 (18%)	0.23
Multiple LOS episodes	10 (26%)	3 (11%)	0.14
Polymicrobial	5 (13%)	1 (4%)	0.13
Gram (+)	5 (13%)	3 (12%)	0.92
CoNS	18 (46%)	14 (52%)	0.65
Gram (−)	13 (33%)	11 (42%)	0.42
Fungi	15 (39%)	1 (4%)	<0.001
CLABSI	10 (30%)	6 (22%)	0.39

EOS: early-onset sepsis; LOS: late-onset sepsis; CoNS: *coagulase*-negative *Staphylococcus*; CLASBI: central-line associated bloodstream infection.

## Data Availability

The dataset is available upon request from the authors.
